# International support for abortion education in medical schools: results of a global online survey to explore abortion willingness, intentions, and attitudes among medical students in 85 countries

**DOI:** 10.3389/fgwh.2024.1253658

**Published:** 2024-03-11

**Authors:** Eglė Janušonytė, Tamara Fetters, Gabriela Cipriano, Iheb Jemel, Cecilia Espinoza

**Affiliations:** ^1^ Faculty of Medicine, Vilnius University, Vilnius, Lithuania; ^2^ Ipas, Chapel Hill, NC, United States; ^3^ Former Assistant Americas Region, International Federation of Medical Students’ Associations, Universidad Peruana Cayetano Heredia, Lima, Peru; ^4^ Faculty of Medicine, University of Monastir, Monastir, Tunisia

**Keywords:** abortion, medical education, medical students, international, survey, postabortion care

## Abstract

**Introduction:**

Access to safe abortion has been recognized as a fundamental human right and important public health priority. Medical schools provide a rare opportunity to expose medical students to comprehensive sexual and reproductive health (SRH) topics and normalize abortion care early in a physician's career.

**Methods:**

This cross-sectional descriptive study used an online survey to explore abortion content in medical curricula and medical student intentions, attitudes, and beliefs regarding abortion provision among 1,699 medical students from 85 countries.

**Results:**

Results demonstrate positive attitudes towards abortion provision, with 83% reporting that “access to safe abortion is every woman's right”. Students also reported a relatively high willingness to provide abortion professionally despite few opportunities to learn about this care. Only one-third of students surveyed reported having taken a gynecology course (*n* = 487; 33%); among these, one-third said they had no content on abortion care in their programs thus far (*n* = 155; 32%), including instruction on postabortion care. Among the two-thirds of students who had some content on abortion care (*n* = 335), either on induced abortion, postabortion care (PAC), or both, 55% said content was limited to one lecture and only 19% reported having an opportunity to participate in any practical training on abortion provision. Despite most students having no or very limited didactic and practical training on abortion, 42% intended to provide this care after graduation. Three-quarters of student respondents were in favor of mandatory abortion education in medical curricula.

**Discussion:**

The findings of this study offer new evidence about abortion care education in medical curricula around the globe, indicating that there is no lack of demand or interest in increasing medical knowledge on comprehensive abortion care, merely a lack of institutional will to expand course offerings and content.

## Introduction

In the last 25 years, access to safe abortion has been widely recognized as a fundamental human right and an important public health priority (1–5). Annually, over 25 million unsafe abortions are performed, resulting in preventable deaths and myriad morbidities (trauma, hemorrhage, sepsis) and the fourth leading cause of maternal death (6, 7). Access to legal abortion is essential to reducing the global burden of unsafe abortions and the number of countries easing restrictions on abortion for their citizens has continued to expand in recent decades in response to increasing evidence to this point (8). According to the Center for Reproductive Rights, nearly 50 countries have liberalized their abortion laws in the past 25 years (9). Nevertheless, policy trends have not created the necessary expansion of services and people around the world still face challenges ensuring and accessing their sexual and reproductive health care, primarily safe abortion care (4).

Access to high-quality, comprehensive abortion care depends largely on the prevalence of skilled providers within the health care system for those who may choose to seek care in health facilities. In many settings where legal indications of abortion are limited and provider stigma and negative attitude around abortion prevail, large groups of women and girls—for example, rural, less educated, poor, adolescent, or unmarried women—experience even greater risk of unsafe abortion (4, 10, 11). Furthermore, implementation challenges in scaling safe abortion practices across already stretched healthcare systems, particularly in resource-poor settings, may further limit access to timely care (12).

Education on medical abortion during medical schools provides an opportunity to counter the stigma faced by abortion providers later in their careers by exposing students to and normalizing comprehensive sexual health care early in a physician's career (13, 14). The hostile environment for abortion globally is a deterrent to educational investment in abortion education as well as individual choices to pursue a professional path that includes abortion provision. A medical education diverse in content and experience can prepare physicians to understand the value sexual and reproductive health care and potentially overcome regulatory or attitudinal obstacles which limit the availability of trained abortion care providers in many countries (12–14).

Presently, formal education on abortion and reproductive rights is scant throughout the United States and Canada, even among residency and specialized programs in obstetrics and gynecology (15, 16). Research on the content, quality, and extent of abortion education internationally is nonexistent, and limited to a small number of national studies which confirm the paucity of abortion educational opportunities found in North America (15–20). Although no internationally comparative data exist, it is reasonable to assume that students receiving training in more legally restrictive settings, more often in the Global South, have even fewer opportunities to learn about safe abortion care and access the most recent evidence and guidance.

Among primary care physicians from the United States, knowledge of abortion is mixed which leads to a gap in institutional provision of adequate baseline medical training in safe abortion (15). There are additional barriers that contribute to gaps in abortion knowledge, such as fear of stigma and personal biases that medical providers may hold prior to entering medical programs, which could be addressed with adequate medical education in abortion during formative years (18, 19). Early exposure to abortion knowledge, issues and exposure to reproductive health and rights can contribute to enhancing medical students' skills in this topic and potentially debunking potential stigma and biases medical student might possess (21–28). Evidence and human rights-based training of health care professionals have become two synchronous components of ensuring access to safe abortion (4). While global evidence around the morbidity and mortality related to unsafe abortion and the impact of safe and legal abortion has continued to expand, little attention has been paid to the human resource requirements such as medical education in abortion topics during formative years, of turning policy changes into practice for the 56,000,000 people who have abortions each year (8).

This study explores international medical students' attitudes towards abortion and abortion education and identifies gaps in institutional curricula in abortion education. The findings of this study offer new evidence about comprehensive reproductive health care education, including abortion care, in medical curricula. The survey also provides insights and recommendations for medical students and educators about their needs and desires to improve their educational content on abortion care and their own decisions about whether they would be willing to provide abortion care post-graduation.

## Methodology

Ipas is an international non-governmental organization that collaborates with a range of partners to develop clinical curricula and train health-care workers to provide induced abortion care and care for complications of unsafe abortions (29). In collaboration with the International Federation of Medical Students' Associations (IFMSA), we conducted an online survey of abortion willingness, intentions, and attitudes among international medical students. IFMSA is a student-led network with 130 national member organizations that represents over 1.3 million students worldwide (30).

This cross-sectional descriptive study used an online quantitative survey fielded from June-October of 2018, to explore the sexual and reproductive health content in the medical curriculum and medical student intentions, attitudes, and beliefs regarding abortion provision in their careers among international medical students. The study used purposive sampling to reach medical students in the IFMSA global and African, the Americas, Eastern-Mediterranean, European and Asia-Pacific regional networks.

The online survey questionnaire (available in English, Spanish and French) was shared with medical students via the IFMSA global and regional medical student mailing lists as well as social media channels. Individuals were asked about policies, attitudes, and practices related to abortion and their abortion content in medical school. All potential respondents on the IFMSA mailing lists were sent a link to an online survey using Survey Monkey software. All medical students registered with the IFMSA in one of their global or regional lists with valid email addresses received an invitation to participate in the online survey via email. IFMSA student members were also able to share the survey link with non-registered students, primarily via social media. Respondents did not have to be a member of the IFMSA (30% of respondents reported that they were not members) but did need to be in medical school. The IFMSA general membership list at the time included over 11,000 students internationally with geographic and topical lists, such as the sexual and reproductive health and rights membership list.

The research protocol and informed consent process were reviewed and approved by the Allendale IRB in the United States. Respondents were not compensated in any way for their participation. Participation was voluntary and all participants were asked to read and agree to the online consent form explaining the study, partners, key personnel, and ethics approval before they moved on to the survey. The benefits to the respondents were explained as being only for the purposes of a more global understanding of medical school curricula and content relating to sexual and reproductive health and abortion. The online survey was anonymized and did not collect the participants' names, IP addresses, email addresses or other identifiable information. Survey content included sections on the socio-demographic information of the medical student; questions on student willingness and intentions to provide abortion care in the future; sexual and reproductive health (SRH) and abortion content of their current medical programs; personal attitudes, beliefs, and experiences with abortion; and questions on participation in IFMSA and other SRH organizations. The survey was circulated and pilot-tested by IFMSA study team members to review content and functionality prior to the general launch.

Descriptive bivariate statistics were used to explore the data. Data were exported from the online survey database and read into Stata statistical software (v. 14) for data cleaning and analysis. Descriptive statistics were used to explore attitudes, willingness to learn about and intentions to provide abortion care post-graduation.

## Results

### Demographics

A total of 1,699 medical students from 85 countries around the world responded and consented to participate in the online survey, of which 1,601 completed the demographic section (Table 1). Sixty-seven percent of respondents were women and the majority, 87%, were under 25 years of age. Respondent geo-locations ranged, with the largest contingent from the European Region (46.7%) followed by Asia Pacific (28.5%), Africa (10.2%), and the Americas (10.2%). Over 60% of participants had over 3 years of medical education at the time of the survey, and only 22% were specializing in obstetrics and gynecology. Students were also asked to describe their personal experience with abortion: 61% reported knowing someone who has had an abortion and 13% knew of someone who had severe abortion-related complications, including death.

**Table 1:** Demographics of medical student respondents (*N* = 1,601).

	*n*	%
Sex		
Female	1,131	70.6%
Male	460	28.7%
Non-binary	10	0.6%
Age		
17–19	270	16.9%
20–24	1,119	69.9%
25–29	186	11.6%
30–41	26	1.6%
Region of medical school attendance		
Europe	747	46.7%
Asia Pacific	456	28.5%
Africa	163	10.2%
Americas	163	10.2%
EMR	72	4.5%
Religion		
Catholic	534	33.4%
Agnostic/Atheist	519	32.4%
Non-Catholic Christian	241	15.1%
Muslim	100	6.3%
Hindu	100	6.3%
Other	99	6.2%
Jewish	7	0.4%
Missing	1	0.1%
Years remaining in medical school		
1–3	598	37.4
4–6	1,003	62.6%
Planned medical school specialization		
Obstetrics & gynecology	362	22.6%
Other specialization	1,239	77.4%
IFMSA member		
Yes	796	49.7%
No	513	32.0%
Missing	292	18.2%
Personal experience with abortion		
Student/partner has been pregnant	86	5.4%
Knows someone who has had an abortion	980	61.2%
Knows someone who has died or had severe abortion complications	204	12.7%

Due to the temporal issues of including medical students in all types of international programs, including those who were in different years in medical school, we asked about content in a more conservative manner considering educational content only from students who had taken gynecology to be those most likely to have been exposed to abortion educational content (data not shown). One-third of students surveyed reported having taken one of more courses in gynecology (*n* = 487; 33%); one-third of these students (*n* = 155; 32%), said they had no content on abortion care in their programs thus far, including instruction on postabortion care (PAC) for the medical or procedural management of abortion complications or pregnancy loss. Among the two-thirds of students who reported having had some content on abortion care (*n* = 335), either on induced abortion, PAC, or both types of care, 329 responded about the amount of content, most said content was limited to one lecture (182; 55%) and only 19% (*n* = 63) reported having an opportunity to do any practical training on abortion provision.

### Attitudes towards abortion

Students were asked if they agreed or disagreed with a series of statements related to abortion attitudes (Table 2). Most students agreed that women need access to safe abortion (87%), it is “a good thing that women can obtain safe abortion services” (85%) and that “access to safe abortion is every woman's right” (83%). Three-quarters of respondents were also in favor of mandatory abortion education in medical curricula. However, 60% agreed that there should be an exemption to abortion provision for those with a conscientious objection and only 37% agreed that they would provide abortion on demand for any reason. Only 12% agreed that students with moral objections to abortion should be exempt from lectures pertaining to abortion provision.

**Table 2:** Attitudes towards abortion.

	*n*	Agree	Neutral or Unsure	Disagree
Statements with majority agreement:				
Women need access to safe abortion services	1,334	87%	6%	7%
It is a good thing women can obtain safe abortion services	1,336	85%	8%	7%
Access to safe abortion services is every woman's right	1,336	83%	8%	9%
A woman should have the right to decide for herself whether or not to have an abortion	1,336	80%	10%	10%
Abortion should be included in mandatory medical school curricula	1,335	75%	18%	7%
Health care providers who conscientiously object to abortion should be required to refer patients	1,330	72%	20%	9%
A woman should always have a right to have an abortion in a case of an unwanted pregnancy	1,335	69%	15%	17%
The government should be responsible for providing abortion as part of free public health care	1,336	68%	17%	15%
Health care providers who conscientiously object to abortion should be allowed to refuse to perform abortions	1,334	60%	22%	19%
Statements with no clear majority sentiment:				
I would provide abortion on demand, to anyone who needs one regardless of their circumstances	1,332	37%	29%	34%
I think I would be discriminated against/stigmatized if I provided abortions to women	1,333	31%	27%	42%
Statements with majority disagreement:				
Termination of pregnancy is against my religion	1,334	29%	21%	51%
Termination of pregnancy is against my morals	1,337	21%	18%	62%
If a patient requested an abortion, I would try to discourage her from seeking the procedure	1,336	20%	28%	52%
Students with moral objections should be excused from lectures about the public health impact of abortion	1,337	12%	23%	65%
I would never perform an abortion under any circumstances	1,334	12%	19%	69%
I would never refer a woman for an abortion under any circumstances	1,332	10%	18%	73%
Abortion should not be provided for any reason	1,331	8%	11%	82%

### Willingness to provide abortion

Participants were asked to consider their willingness to provide abortion under several different scenarios typical for abortion patients (Table 3). A risk to a patient's life (95.7%) or health (91.6%) were among the top scenarios in which these students were willing to provide an abortion, followed by rape (86.6%) or fetal abnormality (83.8%). However, survey respondents seemed to feel less comfortable and were less willing to provide an abortion for patients' personal non-medical reasons such as being unmarried (45.2%) or risk to continuing education (58.9%). Since the survey tool did not require an answer to each question, discomfort in these more challenging values statements about non-medical reasons for abortion resulted in both lower proportions of positive responses and lower levels of overall responses (or more people skipping the uncomfortable questions), rather than explicitly responding that they would not provide abortion under that circumstance. Of the 1,501 participants who responded to the question, 42% stated that they intended to provide abortion services, while 29% were unsure and 25% said that they did not plan to provide this care.

**Table 3:** Willingness & intention to provide abortion.

	*n*	% Yes
I would be willing to provide abortion if…		
Patient's life is at risk	1,259	95.7%
Patient's health is at risk	1,206	91.6%
Patient has been raped	1,139	86.6%
Confirmed fetal abnormality	1,099	83.8%
Patient is a survivor of incest	1,023	78.2%
Patient cannot afford to have a child	854	65.1%
Patient would have to drop out of school	772	58.9%
Patient is unmarried	594	45.2%
For any reason	552	42.0%
Intends to provide abortion in the future	1,501	42.1%

## Discussion

This study is one of very few to examine attitudes and willingness of international medical students in training to accept abortion education and provide services. Results demonstrate positive attitudes towards abortion provision and a generally high willingness to provide abortion after completing their education, despite limited opportunities for this training reported by those who had taken gynecology courses. These findings reflect similar findings to other studies that have shown that students have an interest in and desire for abortion services training in medical school (14, 19, 21–28) and more positive attitudes about abortion among younger people in general (31, 32). This interest is not limited to students planning to specialize in obstetrics and gynecology, who comprised less than a quarter of those surveyed, and who have been shown to have more positive attitudes towards abortion, often due to their personal or professional experiences. Our study shows that personal experiences regarding abortion, such as knowing someone who has had an abortion or someone who has suffered or died from an unsafe abortion, are also common and shared experiences, frequent among medical students that could also be influencing their attitudes.

This study highlights the potential for leveraging support early in health care professionals' careers by offering training at the institutional level. Nearly half of all students in this survey stated their intention to provide abortion services despite a recent international survey of medical schools reporting that nearly 60% of 143 medical faculties represented stating that their schools provided less than nine hours in total of content on sexual and reproductive health and rights (33). Students in this survey reported even less exposure to abortion content, over half of those who had taken gynecology said they only had one lecture on either induced abortion or postabortion care. While the intention to provide abortion services may not result in actual provision due to a range of individual, academic and structural choices, it is unlikely that students deeply opposed to (or uninterested in) abortion would respond with interest and intention to provide abortion care in the future. Research has shown that exposure to abortion training during education may increase the likelihood that student proponents of abortion would be willing to offer abortion services as part of their practice once they have matriculated (13, 14, 24, 26–28, 34–37). Clinical experience of abortion care can help combat stigma against providing abortion services, whether during medical school or during future careers (38). Other studies point to an interest amongst practicing physicians to increase their overall knowledge of abortion provision, which also could have been met through improved medical school options and content related to abortion care and sexual and reproductive health and rights in general (13, 14, 24, 26–28, 36–38).

Medical students in this study reported being open to abortion content as part of their medical training, even those who were not planning to specialize in obstetrics and gynecology. This points to the need to strengthen institutional support for abortion education as a means not only of increasing skilled labor in the health system workforce, but also decreasing stigma by normalizing abortion as part of an integrated medical education. Integrating abortion care into medical training programs could greatly increase access to timely quality abortion for women around the world in a time of expanding political threats to these services (5, 39). This is especially critical in contexts where legalization is recent, to address time required to change social norms, educational curricula in training institutions and build support within the health system to orient new abortion providers (32).

According to an evaluation of Medical Students for Choice's internship program with safe abortion care, authors found that early clinical experiences with abortion and family planning significantly influenced the students' knowledge, attitudes, intentions to provide abortions and ability to counsel patients (36). In another survey of over 800 final-year midwifery students conducted in Ghana, predictors associated with a greater likelihood of providing comprehensive abortion care included having been exposed to multiple forms of education around surgical abortion (37).

These results should be viewed in light of potential limitations in the data. Primary among these is the prevalence of missing data for certain questions, since surveys were conducted online without mandatory questions, some respondents may have skipped questions that made them uncomfortable. Additionally, there are certain biases inherent in our online study design. It is possible that respondents with more sympathetic views about sexual and reproductive health and abortion may have been more likely to respond, introducing social desirability and respondent bias. Furthermore, we did not confirm that respondents were medical students in the online survey, believing that the benefits of a larger and more diverse sample outside of the core, executive or SRH groups of the IFMSA would reduce desirability bias, and was preferable to the possibility that we might have “imposter” students. Since there was no incentive to participate in the survey, we felt that adding additional controls and complexity to ensure that the respondents were students would be a significant deterrent, raising multiple barriers to participation and introducing more challenging confidentiality issues. Another possible limitation is that the responses from students involved in IFMSA may not represent the general population of global medical students. Yet the geographic diversity of our sample, the widespread promotion of the survey and response to the survey through multiple channels and social media, and large sample size were employed to mitigate some of this possibility of these biases. While this survey is not generalizable to all medical students globally, since there are no larger global federations of medical students that are not issue-based, this survey offers a rare opportunity to examine views of this unique and important student group. These limitations were accepted as a risk to utilize the expansive international network of the organization.

As the number of abortion providers continues to decline in the United States (39), and likely globally, due to the hostile environment, stigma, and the undervaluing of comprehensive abortion care, it is crucial that new and creative mechanisms for improving the skills and number of new abortion providers to enable access to one of the most common medical procedures in the world. This study provides evidence that there is no lack of demand or interest in increasing medical knowledge on comprehensive abortion care but merely a lack of institutional will to expand course offerings and content on sexual and reproductive health and rights.

## Data Availability

The raw data supporting the conclusions of this article will be made available by the authors, without undue reservation.
